# Modern Potentiostat Architectures for Electrochemical Sensing: Design, Integration, and Future Directions

**DOI:** 10.3390/mi17060635

**Published:** 2026-05-22

**Authors:** Reagan Aviha, Gymama Slaughter

**Affiliations:** 1 Center for Bioelectronics, Old Dominion University, Norfolk, VA 23508, USA; 2 Department of Electrical and Computer Engineering, Old Dominion University, Norfolk, VA 23508, USA

**Keywords:** electrochemical sensing, potentiostat, wearable biosensors

## Abstract

Potentiostats are essential to electrochemical sensing, enabling precise control of electrode potentials and measurement of current responses. As demand grows for portable, wearable, and point-of-care systems, potentiostat design has evolved from benchtop instruments to compact, low-power, and wirelessly connected platforms. This review provides a comprehensive, system-level perspective on modern potentiostat architectures, covering operational principles, analog front-end design, signal generation and acquisition, communication protocols, and software integration. Unlike prior reviews that treat these aspects independently, this work integrates electrochemical theory with electronic design and data communication frameworks. Key components, including operational amplifiers, transimpedance amplifiers, DAC/ADC subsystems, and microcontroller-based control, are examined alongside communication protocols such as SPI, I^2^C, Bluetooth Low Energy, Wi-Fi, and NFC. Critical challenges related to miniaturization, noise, power constraints, and reproducibility are analyzed using representative platforms. This review highlights the transition of potentiostats into integrated, intelligent, and connected sensing systems, and outlines design considerations for scalable electrochemical applications in clinical, environmental, and industrial domains.

## 1. Introduction

The prevalence of chronic diseases has increased significantly in recent decades, driven in part by delayed diagnosis and limited access to affordable testing methods [1,2,3]. Diabetes mellitus is a prominent example, with a growing population of diagnosed and undiagnosed individuals worldwide [4,5]. The disease is characterized by persistent hyperglycemia, which can lead to complications including nephropathy, retinopathy, cardiovascular disease, and neuropathy if not properly managed [6]. These complications impose substantial healthcare and socioeconomic burdens, underscoring the need for early diagnosis and continuous monitoring [7,8].

Glucose monitoring remains central to diabetes management. Conventional laboratory-based methods offer high accuracy but are not suitable for continuous or real-time monitoring due to their cost, infrastructure requirements, and lack of portability [9]. To address these limitations, alternative sensing platforms have been developed, including non-enzymatic sensors, which offer improved stability, reduced cost, and greater flexibility compared to enzyme-based systems that are sensitive to environmental conditions such as temperature and pH [10,11]. Non-enzymatic glucose detection can be achieved through optical, mass-based, fluorescent, and electrochemical transduction mechanisms [12]. Among these, electrochemical sensing is widely adopted due to its high sensitivity, low cost, and compatibility with miniaturized and wearable systems [13]. Detection is typically based on redox reactions at the electrode surface, where glucose oxidation generates measurable electrical signals proportional to its concentration [14,15,16]. Accurate signal acquisition requires electronic interfaces capable of controlling the electrochemical environment and converting current responses into quantifiable outputs [17].

The potentiostat is a key component in such systems, enabling precise control of the working electrode potential relative to a reference electrode while measuring the resulting current through the counter electrode [18,19,20]. However, conventional potentiostats are often bulky, power-intensive, and composed of discrete components, limiting their integration into portable and wearable platforms [21]. Advances in microelectronics, particularly complementary metal-oxide-semiconductor (CMOS) technologies, have enabled the development of compact, low-power potentiostats with integrated functional blocks such as transimpedance amplifiers, low-noise current readouts, and programmable biasing circuits [20,21,22,23,24,25]. In parallel, wireless communication and real-time data transmission capabilities have expanded the potential for remote and distributed health monitoring [26].

Despite these advancements, challenges remain in achieving reliable and selective electrochemical detection. Interference from electroactive species such as ascorbic acid, uric acid, and dopamine can distort measurements, while noise and signal drift affect accuracy and stability [27]. These challenges are particularly pronounced in portable and wearable systems, where power and size constraints must be carefully balanced against sensing performance. Although a substantial body of literature exists on electrochemical sensing, potentiostat circuits, and wireless analytical platforms, most studies treat these domains in isolation. Reviews focused on electrochemistry typically emphasize electrode materials, sensing mechanisms, and analytical metrics (e.g., sensitivity, detection limits, selectivity) without addressing circuit-level propagation or system-level constraints. Consequently, practical trade-offs, such as noise, signal stability, and power consumption, are often underexplored. Conversely, reviews centered on potentiostat or analog front-end (AFE) design prioritize amplifier architectures, noise optimization, and ADC/DAC selection, but rarely consider electrochemical signal characteristics and associated artifacts. Similarly, emerging work on wearable and IoT-enabled sensing frequently emphasizes connectivity or application demonstrations, treating sensing and electronics as black boxes rather than deeply integrated subsystems.

This review provides a comprehensive, system-level overview of potentiostat technologies, covering operational principles, circuit architectures, communication strategies, and integration into modern sensing platforms (2020–2025). Particular emphasis is placed on their role in enabling portable, low-power, and real-time electrochemical diagnostics. This review bridges electrochemical operating principles with circuit-level design by explicitly linking electrode behavior—such as capacitance, noise sources, and bias requirements—to analog front-end (AFE) design, stability considerations, transimpedance amplifier architecture, and ADC selection. It further treats communication frameworks (Bluetooth Low Energy, Wi-Fi, and cloud connectivity) as integral components of sensing systems rather than peripheral add-ons. The associated trade-offs in resolution, bandwidth, power consumption, and noise are examined in the context of portable and wearable potentiostats. By integrating these perspectives, the review connects classical instrumentation with modern system-on-chip architectures, illustrating the transition from discrete potentiostats to decentralized, wearable sensing platforms. Additionally, it contextualizes emerging trends, such as edge computing and cloud-assisted analytics, within practical hardware and system-level constraints.

## 2. Historical Evolution of Potentiostats

The development of potentiostats originated from the need to precisely control and measure electrochemical processes. Early electrochemical studies established that reaction behavior is governed by electrode potential rather than current, as described by the Nernst equation (1889), which relates redox equilibria to electrode potential [28,29]. Initial experimental approaches, including those reported by Ostwald in the late 19th century, relied on manual circuit balancing to maintain a constant working electrode (WE) potential relative to a reference electrode (RE), typically a calomel electrode [30,31]. These methods established the theoretical basis of potential control but were inherently limited in precision, reproducibility, and scalability due to manual operation. The introduction of polarography by Jaroslav Heyrovsky in 1922 marked a major advancement, enabling systematic current–potential measurements using a dropping mercury electrode (DME) [32,33]. While this technique improved analytical capability and reduced measurement time, it primarily relied on two-electrode configurations in which the counter electrode (CE) also functioned as the reference, limiting accurate potential control.

A significant milestone was achieved in 1942 with the development of the three-electrode potentiostat by Archie Hickling, which enabled independent control of WE potential while measuring current at the CE [34]. The incorporation of negative feedback mechanisms allowed stable and reproducible potential regulation, forming the basis for modern electrochemical techniques such as voltammetry [35,36]. The transition from vacuum tube electronics to solid-state transistor-based systems further improved stability, reduced noise and drift, and enhanced temporal resolution [37,38]. These developments supported the widespread adoption of techniques such as cyclic voltammetry (CV) and chronoamperometry (CA), which remain central to electrochemical sensing. Further advances in electronics and computation during the 1970s and 1980s facilitated the integration of operational amplifiers and modular circuit architectures, leading to the commercialization of laboratory potentiostats [39,40]. The introduction of microprocessors and digital control systems in the late 1980s and 1990s enabled automated waveform generation and real-time data acquisition, significantly improving measurement consistency [41,42].

**Figure 1:**
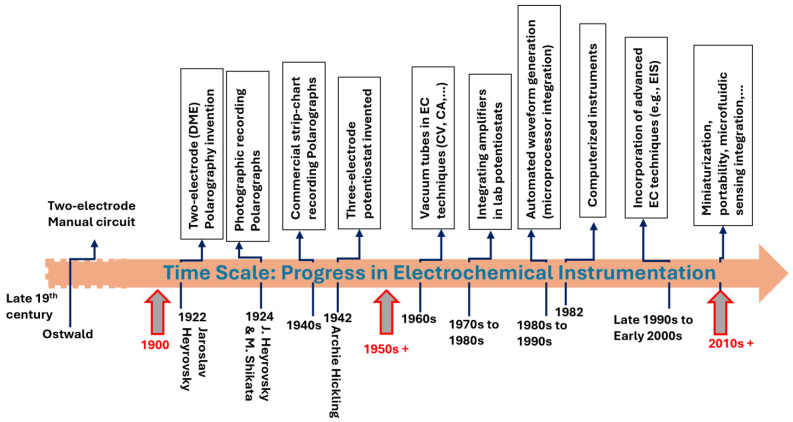
Historical progression of potentiostatic electrochemical instrumentation, illustrating key technological milestones from early manual potential control to modern digital, miniaturized, and integrated sensing platforms.

In the late 1990s and early 2000s, digital potentiostats incorporating advanced techniques such as electrochemical impedance spectroscopy (EIS) expanded the scope of electrochemical analysis by enabling detailed characterization of interfacial processes and charge-transfer dynamics [43,44,45]. More recent developments since the 2010s have focused on miniaturization, portability, and integration with modern sensing platforms. Advances in embedded systems, microcontrollers, and wireless communication have enabled low-cost potentiostats suitable for point-of-care diagnostics and field applications [21,46,47], while maintaining performance comparable to laboratory instruments. Modern potentiostats increasingly incorporate real-time data processing, cloud connectivity, and integration with microfluidic and wearable platforms [48,49,50]. This evolution has transformed potentiostats from standalone laboratory instruments into integral components of connected sensing systems used in biosensing, lab-on-a-chip technologies, and real-time monitoring applications [28,51,52]. This progression reflects a shift from fundamental potential control toward integrated, system-level electrochemical sensing platforms. A summary of key milestones in potentiostat development is presented in Figure 1.

## 3. Principle of Potentiostat

**Figure 2:**
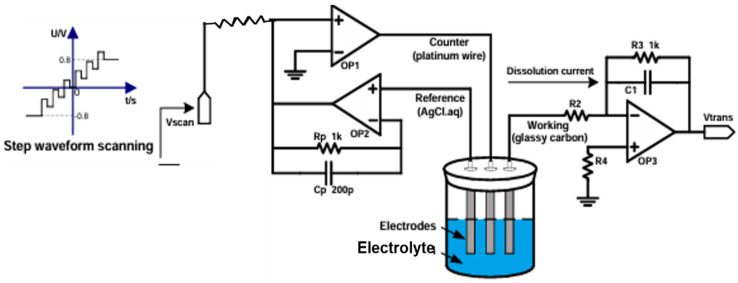
Schematic of a potentiostat architecture illustrating how an excitation signal is applied to regulate the working electrode potential relative to the reference electrode, while the resulting current is measured through the counter electrode [54].

The primary function of a potentiostat is to precisely control the potential of the WE relative to a RE while measuring the resulting current flowing through the CE. In early two-electrode systems, the measured response was influenced by coupled potential and current variations, limiting control and interpretability. This limitation was addressed by the introduction of the three-electrode configuration, where the RE provides a stable potential independent of current flow [34]. In a typical potentiostat architecture, the RE is connected to a high-input-impedance operational amplifier (OPA), ensuring negligible current flow and preserving its stability [53]. The applied excitation signal, generated by a digital-to-analog converter (DAC), defines the desired WE–RE potential difference. When the electrochemical cell is connected (Figure 2), redox reactions at the WE perturb this potential, and the control circuitry adjusts the current through the CE to maintain the commanded voltage through closed-loop feedback [54].

Most potentiostat designs employ multiple OPAs with distinct roles, including a control (error) amplifier, a reference buffer, and a transimpedance amplifier (TIA). The control amplifier compares the applied voltage with the sensed WE potential and regulates the CE current accordingly. The TIA converts the resulting faradaic current into a measurable voltage, typically determined by the feedback resistor (e.g., R3 = 1 kΩ in Figure 2), which defines sensitivity and measurement range [55]. The output is subsequently digitized using an analog-to-digital converter (ADC). Accurate potentiostatic operation depends on maintaining RE stability and minimizing drift. Since the RE is highly sensitive to loading effects, buffering circuits are used to isolate it from the rest of the system [56]. The control amplifier must exhibit high gain and stability to ensure that the WE potential closely follows the applied command signal. Modern potentiostats also incorporate compensation strategies to address non-ideal behaviors such as double-layer capacitance, charge-transfer resistance, and diffusion effects, which introduce nonlinearities in techniques such as CV, CA, and EIS [57].

The electrochemical behavior of the system is governed by thermodynamic and kinetic relationships. Under equilibrium conditions with no net current flow, the electrode potential is described by the Nernst equation [58]:

(1) $$E=E^{o}-\frac{\mathrm{RT}}{\mathrm{nF}}\ln(\frac{a_{ox}}{a_{\mathrm{red}}})$$

where E^o^ is the standard potential, R is the universal gas constant, T is the absolute temperature, n is the number of electrons transferred, F is Faraday’s constant, a_ox_ and a_red_ represent the activities of oxidized and reduced species, respectively.

When current flows under potentiostatic control, the system operates under non-equilibrium conditions and is described by the Butler–Volmer equation [59]:

(2) $$j=j_{o}[e^{\beta }-e^{\alpha }]e^{(E-E_{\mathrm{eq}})E/\mathrm{RT}}$$

where j is the faradaic current density, j_o_ is the exchange current density, E_eq_ is the equilibrium potential, and α and β are charge-transfer coefficients. These relationships link the applied potential to measurable current responses, forming the basis for quantitative electrochemical sensing.

**Figure 3:**
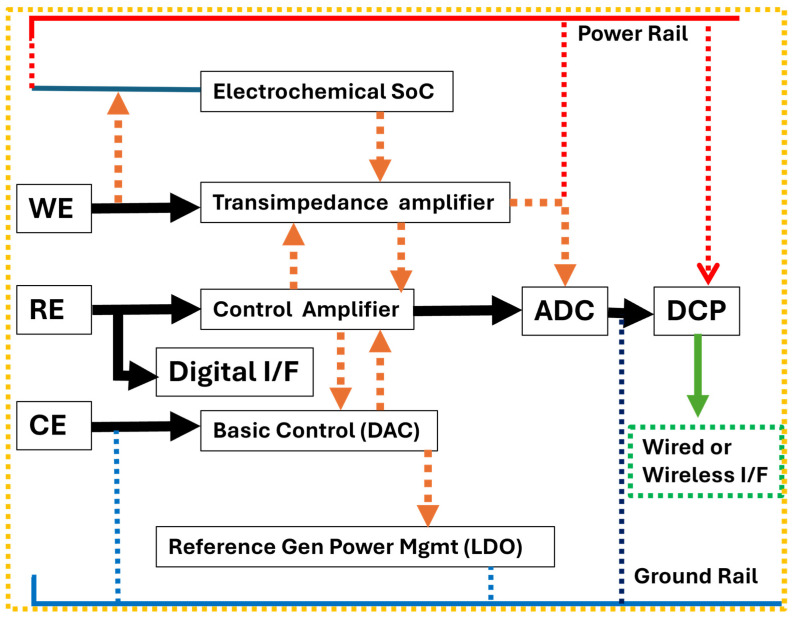
Schematic illustration of the potentiostat integrated on a modern System-on-Chip.

Overall, potentiostatic operation relies on the coordinated interaction of closed-loop feedback control, high-impedance reference buffering, and current-to-voltage conversion, coupled with thermodynamic and kinetic models. This integration enables precise potential regulation, high-sensitivity current detection, and accurate interpretation of electrochemical signals, which are essential for quantitative sensing in complex environments. As illustrated in Figure 2, this architecture highlights the interplay between control and measurement pathways. Modern potentiostats are integrated into a system-on-chip (SoC) strategy where all their functions are consolidated. This can be illustrated by the scheme in Figure 3.

## 4. Components of Potentiostats

Electrochemical reactions are governed by the relationship between electrode potential and current. Accurate control of potential and measurement of current are essential for investigating reaction kinetics, thermodynamics, and mass transport phenomena [60]. A potentiostat maintains a programmed potential difference between electrodes while monitoring the resulting current through a closed-loop feedback system that compensates for variations in solution resistance, electrode surface condition, and reaction dynamics [61]. Potentiostat performance is determined by the coupled interaction of feedback-controlled potential regulation, electrochemical cell configuration, and electrode properties. Together, these factors govern signal stability, sensitivity, response time, and measurement accuracy under both ideal and non-ideal conditions.

### 4.1. Electrochemical Cell Interface

Although external to the instrument, the electrochemical cell strongly influences potentiostat performance. Its design is determined by the intended application and includes parameters such as electrode size (typically 1–10 cm), electrolyte concentration (medium to high), volume (100 mL to several liters), and electrode geometry [62]. Most systems employ a three-electrode configuration to decouple potential control from current flow [63]. Common configurations include parallel electrode arrangements and concentric geometries in which the CE surrounds the WE. Application-specific designs are used for corrosion, cyclic voltammetry, and analytical measurements. For example, corrosion cells require chloride-free crevice regions to avoid parasitic reactions [64], while cyclic voltammetry cells are optimized for small volumes with dimensions typically ranging from 30 to 60 mm in diameter and 25 to 50 mm in height [65]. Cell geometry and electrolyte configuration influence solution resistance and mass transport, directly impacting response time, signal fidelity, and reproducibility.

#### 4.1.1. Working Electrode

The WE is the site of the electrochemical reaction and is controlled relative to the RE. Materials such as platinum, gold, glassy carbon, and graphene-based electrodes are commonly used, depending on the application [66]. The geometry and positioning of the WE relative to the CE determine current distribution and response dynamics. For instance, shorter distances between WE and CE improve response time, while electrode shape (e.g., rod or planar) influences current uniformity [67,68]. The WE defines the sensing interface, where its material properties, surface chemistry, and geometry directly determine sensitivity, selectivity, and temporal response of the electrochemical system.

#### 4.1.2. Counter Electrode

The CE completes the electrical circuit by supplying or sinking current required to maintain the WE potential. It is typically fabricated from inert materials with large surface area, such as platinum mesh or wire, to minimize polarization effects [69,70]. The CE geometry is chosen to complement the WE configuration to ensure uniform current distribution [67,71]. In systems where CE reactions produce interfering species, membranes or frits may be used to isolate the CE compartment [72]. Proper CE design prevents current limitation and unwanted side reactions, ensuring that measured signals originate primarily from processes at the WE.

#### 4.1.3. Reference Electrode

The RE provides a stable and well-defined potential against which the WE is controlled. It must operate under negligible current to avoid potential drift [73]. Common RE types include Ag/AgCl and SCE [74]. Practical considerations include shielding the RE from gas bubbles and minimizing diffusion effects through proper positioning and separation from the WE [75]. Use of capillary interfaces or frits reduces solution resistance, but excessive resistance can introduce delays exceeding the potentiostat response time [76]. Additionally, improper placement may lead to signal distortion caused by local concentration gradients or gas accumulation. Stability and positioning of the RE are therefore critical for maintaining accurate potential control.

**Figure 4:**
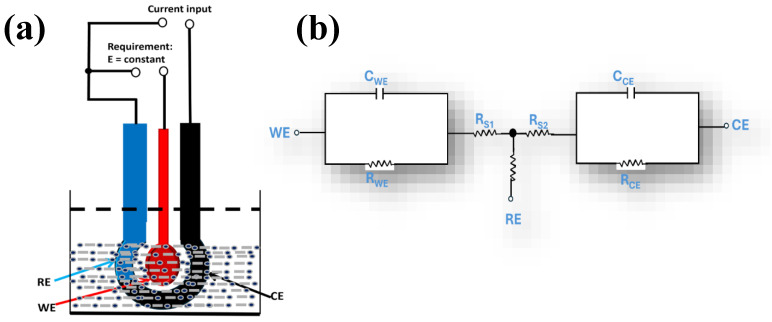
(**a**) Schematic representation of a three-electrode electrochemical cell illustrating the working, reference, and counter electrodes, and (**b**) the corresponding equivalent circuit model describing solution resistance, charge-transfer resistance, and double-layer capacitance.

The electrochemical cell in Figure 4a can be represented by an equivalent circuit, as shown in Figure 4b, where double-layer capacitances (C_CE_, C_WE_), charge-transfer resistances (R_RE_, R_WE_, R_CE_), and solution resistances (R_S1_, R_S2_) describe the system behavior [77]. The faradaic contributions at the CE are typically small and can often be neglected. Equivalent circuit modeling enables separation of resistive and capacitive contributions, allowing more accurate interpretation of electrochemical signals in sensing applications. This interaction between circuit control and electrochemical interface defines the ultimate performance limits of potentiostatic sensing systems.

### 4.2. Operational Amplifier in Potentiostatic Circuit

**Figure 5:**
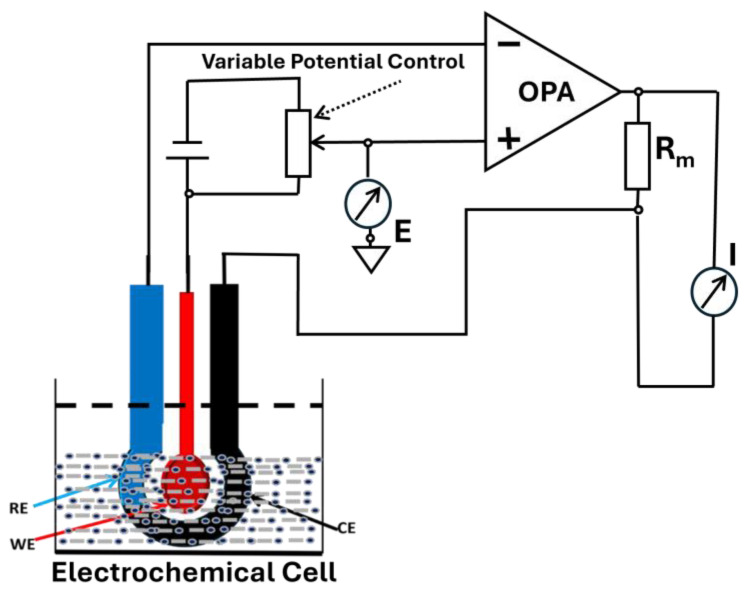
Schematic of a single-operational-amplifier potentiostat illustrating basic closed-loop control used to maintain the working electrode potential relative to the reference electrode in an electrochemical cell.

Operational amplifiers form the core of potentiostatic circuitry, enabling precise control of electrode potential and accurate measurement of electrochemical current. An OPA consists of inverting (−) and non-inverting (+) inputs and an output terminal, and operates with high gain and high input impedance to minimize perturbations at sensitive nodes such as the reference electrode (RE) [18]. In its simplest implementation, a potentiostat can be realized using a single OPA (Figure 5), where negative feedback forces the potential difference between the inverting and non-inverting inputs toward zero. When the electrochemical cell is connected, deviations in the WE potential relative to the RE are corrected by adjusting the current through the CE, thereby maintaining the desired potential [78,79].

**Figure 6:**
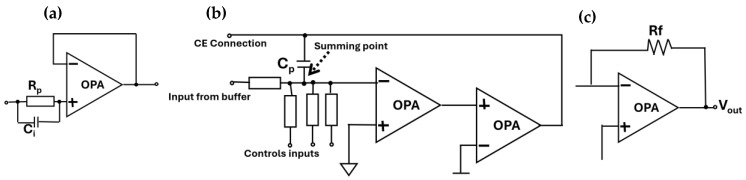
Operational amplifier configurations employed in potentiostatic circuits: (**a**) buffer for high-impedance isolation of the reference electrode, (**b**) summing amplifier for combining multiple input signals, and (**c**) transimpedance amplifier (TIA) for converting electrochemical current into a measurable voltage.

Practical potentiostat designs employ multiple OPAs configured for distinct roles, including buffering, control, signal summation, and current-to-voltage conversion. A buffer (voltage follower), as shown in Figure 6a, is used at the RE to isolate its high-impedance node (10^11^–10^14^ Ω) and extremely low input current (pA range), preventing loading effects and preserving measurement stability [80]. Protection components such as resistors (Rp) and capacitors (Ci) are incorporated to mitigate transient disturbances and enhance circuit stability.

The control amplifier maintains the WE potential at a preset value relative to the RE using a negative feedback loop. The applied command voltage is continuously compared with the sensed RE potential, and the resulting error signal drives the CE to correct any deviation. This configuration compensates for resistive and capacitive effects in the electrolyte, ensuring accurate potential regulation under dynamic conditions [81]. In applications requiring complex excitation signals, multiple voltage inputs may be combined using a summing amplifier (Figure 6b). This enables superposition of direct current (DC) bias with alternating current (AC) signals, allowing controlled perturbation of the electrochemical system across multiple time and frequency domains [82]. Measurement of electrochemical current is achieved using a transimpedance amplifier (TIA) (Figure 6c), where the WE is connected to the inverting input, creating a virtual ground condition. The TIA converts electrode current into a voltage according to

(3) $$V_{out}=-I_{WE}\times Rf$$

where *I_WE_* is the current at the WE and *Rf* is the feedback resistor [83]. The choice of *Rf* determines the sensitivity and dynamic range of the measurement. In addition to the feedback resistor (*Rf*), small series resistors can be introduced between the electrode and the TIA to enhance stability by forming a compensating RC network that mitigates phase lag within the circuit. This can be useful in ensuring the low-noise design and minimal input bias current remain essential for detecting signals in the picoampere range. OPA performance is frequency-dependent; gain decreases and phase shift increases with frequency, which can lead to instability and oscillation when phase approaches 180°. An operational amplifier (OPA) with a gain–bandwidth product exceeding the signal bandwidth can improve TIA stability and transient response. However, increased bandwidth introduces additional noise, reducing the signal-to-noise ratio (SNR). Therefore, OPAs with appropriate internal compensation for capacitive loads are preferred, particularly in low-current electrochemical measurements. Compensation techniques, such as capacitive feedback, are therefore required to maintain stable operation across the desired bandwidth [84,85]. Incorporating a feedback capacitor across the feedback resistor (Rf) introduces a dominant pole that limits TIA bandwidth and modifies the phase margin, thereby improving stability. Proper selection of this capacitor shifts the onset of potential instability, ensuring stable current-to-voltage conversion. The choice is usually made such that

(4) $$f_{p}\approx \frac{1}{2\pi R_{f}C_{f}}\;$$

where *C_f_* is the chosen capacitor.

While high-bandwidth OPAs can be advantageous, their selection should be guided by the minimum bandwidth requirements of the specific electrochemical technique. In many cases, narrow-bandwidth operation improves noise performance and system stability without compromising relevant signal content, particularly in amperometric and potentiometric measurements. Moreover, potentiostat performance relies on the coordinated operation of high-impedance buffering, closed-loop feedback control, signal conditioning, and current-to-voltage conversion. Together, these functions enable stable potential regulation, high-sensitivity current detection, and reliable measurement under dynamic and non-ideal electrochemical conditions. These circuit-level functions directly define the limits of sensitivity, stability, and temporal resolution in electrochemical sensing systems.

### 4.3. Signal Generation

Potentiostatic electrochemical measurements are initiated by an excitation signal generated using a DAC. This signal defines the applied potential waveform (e.g., sinusoidal, triangular), which is used to probe electrochemical responses [86]. The fidelity of this excitation waveform, its amplitude, timing, and shape directly determines the accuracy of the measured electrochemical response [43]. The DAC converts digital commands from a microcontroller or computer interface into a continuous analog voltage, which serves as the reference input to the potentiostat control amplifier, regulating the potential between the WE and RE. Signal quality is governed by DAC parameters such as resolution, update rate, settling time, and integral nonlinearity. The analog output is defined by

(5) $$V_{DAC}=\frac{V_{range}}{2^{B}}\times N_{STEPS}$$

where *V_range_* is the DAC voltage range (typically 0–2.4 V, 3.3 V, or 5.0 V), B is the bit resolution, and N_STEPS_ represents the discrete step index. Since DAC outputs are inherently unipolar, electrochemical techniques such as CV require level-shifting circuits to generate bidirectional potentials. This enables symmetric anodic and cathodic sweeps necessary for full electrochemical characterization. Several DAC architectures are used depending on performance requirements [87]. The binary-weighted DAC is a simple implementation that assigns resistor values in geometric progression (R, 2R, 4R, …) based on bit significance [88]. The output voltage is given by

(6) $$V_{Out}=V_{ref}\left(\frac{b_{N}}{2}+\frac{b_{N-1}}{2^{2}}+\ldots +\frac{b_{1}}{2^{N-1}}+\frac{b_{0}}{2^{N}}\right)$$

where *b_i_*’s represent the binary bit values and *V_ref_* is the reference voltage. For example, a 4-bit input of 0101 yields

$$V_{Out}=V_{ref}\left(\frac{0}{2}+\frac{1}{2^{2}}+\frac{0}{2^{3}}+\frac{1}{2^{4}}\right)=V_{ref}\left(\frac{0}{2}+\frac{1}{4}+\frac{0}{8}+\frac{1}{16}\right)=V_{ref}\left(0+0.25+0+0.0625\right).$$

If an input of 0101 is used on a 4-bit DAC, the conversion will be as follows:

(7) $$V_{Out}={0.3125\;V}_{ref}$$

However, binary-weighted DACs are highly sensitive to resistor mismatch and switching errors, particularly at higher resolutions (>8 bits), resulting in nonlinearity and waveform distortion [89,90]. To address these limitations, the R-2R ladder DAC uses only two resistor values, improving matching and linearity [91]. Although increased ladder depth introduces larger RC time constants that may limit speed, this architecture offers a balance between precision and dynamic performance, making it suitable for fast-scan CV and high-frequency EIS [92]. Sigma-delta DACs are widely used in high-resolution (>20-bit) systems due to their superior linearity and low noise characteristics [93]. They employ oversampling and noise shaping to enhance effective resolution. The resolution and output are defined as

(8) $$\Delta V=\frac{V_{range}}{2^{B}}$$

where Δ*V* is the resolution

(9) $$V_{DAC}=\Delta V\times N$$

where *N* is the voltage step whose total value is $N=2^{B}-1$.

These DACs are particularly suitable for low-frequency and steady-state measurements such as CA and CV, where signal stability is critical. In advanced potentiostat designs, multiple DAC outputs may be combined using a summing amplifier to generate complex excitation signals [94]. This enables precise control of multi-component waveforms required for advanced electrochemical techniques. Overall, the DAC subsystem governs the accuracy, resolution, and temporal fidelity of the excitation signal. The quality of electrochemical perturbation and subsequent influence on the reliability of kinetic and analytical measurements in this architecture is determined by the linearity of bidirectional waveforms. Thus, waveform generation quality sets the fundamental limit on the accuracy of electrochemical interrogation.

### 4.4. Signal Acquisition

Signal acquisition focuses on accurately capturing the electrochemical response at the working WE under an applied potential. The resulting current is converted to a voltage using a TIA and subsequently digitized using an ADC. Prior to digitization, the signal passes through an anti-aliasing stage to suppress high-frequency noise and prevent spectral distortion, preserving signal fidelity [43,95]. The relationship between the WE current and the ADC input voltage is defined by

(10) $$V_{ADC}=I_{WE}\times R_{TIA}$$

where $I_{WE}$ is the electrode current and R_TIA_ is the feedback resistor. The selection of R_TIA_ must ensure that the resulting voltage remains within the ADC input range while maximizing resolution. Matching R_TIA_ to the expected current range optimizes dynamic range and prevents signal saturation or loss of sensitivity. Electrochemical techniques often produce bidirectional (anodic and cathodic) currents, whereas ADCs typically accept unipolar inputs. To address this, level shifting is implemented using a summing amplifier:

(11) $$V_{shifted}=V_{TIA}+V_{\frac{ref}{2}}$$

This transformation maps the signal into the ADC input range (0 to V_ref_), enabling full digitization of both positive and negative current responses [96]. The overall performance of the acquisition chain depends on the combined characteristics of the excitation source, TIA, and ADC, including noise, settling time, and conversion accuracy [93]. Additional buffering may be incorporated to stabilize the signal and reduce dynamic error, particularly in high-frequency measurements. Two common ADC architectures are employed in potentiostat systems: successive approximation register (SAR) and sigma-delta converters. SAR ADCs use a comparator-based approach to iteratively approximate the input voltage relative to an internal reference, providing high-speed conversion suitable for capturing fast transient electrochemical signals required in wearable glucose sensors [97]. In contrast, sigma-delta ADCs employ oversampling and noise shaping to achieve high resolution and improved SNR, followed by digital filtering and downsampling [98]. While sigma-delta converters offer superior sensitivity for low-level signals, their slower conversion rates introduce latency, requiring trade-offs between resolution and temporal response. This consideration is particularly relevant for slow-varying signal platforms, such as open-circuit measurements in wearable systems. A comparison of ADC architectures in terms of power consumption and key performance parameters is provided in Table 1, based on representative integrated microcontrollers and standalone ADCs.

Consequently, accurate electrochemical signal acquisition depends on the coordinated operation of current-to-voltage conversion, signal conditioning, level shifting, and digitization. The trade-off between speed, resolution, and noise performance across the acquisition chain ultimately defines the fidelity and interpretability of electrochemical measurements. Achieving ultra-low current resolution and low noise typically requires high TIA gain, oversampling ADCs, and stable reference circuits, all of which increase power consumption. As a result, wearable systems often prioritize sufficient rather than maximum resolution. Measurement accuracy can instead be preserved through calibration, signal averaging, and adaptive measurement strategies, enabling low-power operation compared to high-powered or intermittently powered benchtop potentiostats. Thus, acquisition chain design sets the fundamental limits on temporal resolution and sensitivity in electrochemical sensing.

### 4.5. Microcontrollers

Microcontrollers coordinate signal generation and acquisition in potentiostat systems by controlling the DAC and ADC. They are compact, integrated computing units that combine a central processing unit (CPU), communication interfaces, and analog/digital peripherals on a single chip, enabling low-power, real-time operation in embedded systems [99,100]. Integration of control, processing, and peripheral functions within a microcontroller reduces system latency, improves synchronization between excitation and measurement, and minimizes external noise sources in compact designs. Microcontrollers enable simplified system architectures by incorporating onboard DAC and ADC modules, as demonstrated in low-cost implementations such as the HuntStat potentiostat for CV and CA measurements [101]. This integration reduces hardware complexity while enabling flexible, software-defined control of electrochemical experiments.

In modern potentiostats, microcontrollers also manage communication and data handling, including user-command reception, real-time visualization, and wireless transmission. For example, ESP32-based systems utilize built-in Wi-Fi modules to support remote monitoring and distributed sensing in point-of-care (PoC) applications [102,103]. The communication layer is tightly coupled with the measurement loop, enabling synchronized waveform generation, ADC sampling, and real-time data transmission [104]. Within the electrochemical control loop, microcontrollers perform key functions such as generating excitation waveforms via the DAC, adjusting gain settings in the TIA, and synchronizing ADC sampling with the applied potential. Pulse-width modulation (PWM) peripherals can also be used to implement PWM-based DACs in ultra-low-cost or wearable systems [105,106]. Accurate timing control ensures precise correlation between applied potential and measured current, which is essential for reliable electrochemical analysis.

The selection of microcontrollers depends on system requirements, including processing speed, memory capacity, peripheral availability, power consumption, and cost [107]. High-performance applications, such as fast waveform generation and computationally intensive signal processing, often utilize 32-bit architectures (e.g., ARM Cortex-M) in platforms such as STM32-based potentiostats [108]. Integration of analog peripherals with digital control enhances portability while maintaining performance [109]. Overall, the microcontroller’s performance directly governs synchronization, timing accuracy, noise susceptibility, and communication efficiency. The balance between computational capability and power consumption ultimately defines system responsiveness, measurement fidelity, and suitability for portable and wearable electrochemical sensing applications. Thus, microcontroller design sets the temporal and computational limits of modern electrochemical sensing systems.

### 4.6. Power Supply and Mains Operations

The power supply is a critical component in potentiostat design, as electrochemical measurements rely on stable, low-noise voltage sources for accurate signal acquisition. Small electrochemical currents, often in the nanoampere range, are highly susceptible to power supply fluctuations and grounding effects [110,111]. Low-noise DC rails are therefore essential to maintain signal integrity. Portable potentiostats commonly use battery power to minimize electrical noise and provide inherent isolation, while benchtop systems employ regulated mains supplies. Modern designs incorporate AC-to-DC converters or switching regulators to generate stable DC voltages, typically combined with analog filtering stages to reduce ripple and switching artifacts [112,113,114].

Electrical isolation is required to prevent ground-loop errors and ensure accurate measurement relative to the reference electrode potential. Isolation techniques decouple the electrochemical cell from external ground references, eliminating unintended current paths that can introduce offset errors and degrade measurement accuracy [115]. Many laboratory potentiostats utilize dual-polarity supply rails (e.g., ±12 V or ±15 V) to support bipolar electrochemical experiments and provide sufficient compliance voltage for driving the counter electrode (CE) [116]. The available bias range must be designed within the limits of the power supply to ensure stable operation, as demonstrated by Ashoori et al. [117]. In contrast, modern integrated and wearable systems often operate using single-supply configurations to reduce power consumption and system complexity.

Noise mitigation strategies include the use of low-noise regulators, inductor–capacitor (LC) filters, and separation of analog and digital ground planes [118]. These approaches are essential for maintaining the stability of sensitive components such as the control amplifier and TIA, which are particularly vulnerable to power-induced disturbances. Proper shielding is also required in mains-powered systems to ensure both signal integrity and user safety [119]. Alternative power sources such as lithium-ion batteries and USB supplies are commonly used in portable systems to reduce ripple and eliminate switching transients. However, applications requiring higher current delivery, such as CA with large electrodes or low-resistance electrolytes, may demand currents on the order of tens to hundreds of milliamperes at the WE [18]. These conditions require power supplies capable of delivering stable current without voltage sag or transient fluctuations. In these applications, power supply design defines the achievable electrochemical operating envelope by setting noise floor, voltage range, and current delivery limits. Effective regulation, filtering, and isolation prevent propagation of electrical interference through the signal chain, while sufficient voltage and current capacity ensure stable control under varying electrochemical loads. Thus, power supply design fundamentally constrains sensitivity, stability, and dynamic range in electrochemical sensing systems.

### 4.7. Filters and Noise Reduction

Electrochemical measurements often involve the detection of very small currents, making them highly susceptible to noise from electronic components, environmental interference, and power supply fluctuations. Thermal noise in resistive elements and fluctuations in bias currents can propagate through the signal chain, particularly affecting the input and first amplification stages, where signal levels are lowest [120]. Low-noise OPAs are therefore essential in potentiostat design. These amplifiers are selected for low input-referred noise, stable gain, and appropriate bandwidth to minimize distortion and parasitic effects. Proper bandwidth limitation reduces high-frequency noise while preserving relevant electrochemical signal components [121].

Analog and digital low-pass filters (LPFs) are widely used to suppress high-frequency noise and improve signal quality. These filters may be implemented in hardware or during post-processing to smooth residual fluctuations and enhance measurement stability [121,122]. The cutoff frequency must be carefully selected to balance noise suppression with signal fidelity, as overly aggressive filtering can distort electrochemical waveforms and lead to misinterpretation of reaction kinetics and transport processes. Advanced noise reduction techniques include chopper stabilization and dynamic element matching (DEM). Chopper stabilization in transimpedance-based amplifier circuits mitigates low-frequency noise by modulating the input signal to frequencies above the 1/*f* noise corner, followed by demodulation to recover the baseline signal while suppressing flicker noise and DC offset. This approach is particularly important for wearable electrochemical sensors, which operate at low bandwidths (<10 Hz) and are susceptible to motion-induced fluctuations. As a result, chopper stabilization enhances long-term signal stability under dynamic physiological conditions. The DEM reduces mismatch-induced errors by redistributing them over time [123]. These techniques are particularly important in low-frequency and steady-state measurements, such as amperometry, where signal components often overlap with noise spectra.

Electromagnetic interference (EMI) can be mitigated through shielding strategies such as grounded enclosures, shielded cables, and Faraday cages. This prevents capacitive coupling from human motion, nearby electronics and communication protocols used. The separation of analog and digital ground planes further reduces coupling of noise into sensitive measurement circuits, which is critical when measuring ultra-low currents [124]. The incorporation of driven shields (active guarding) around the working electrode, TIA, and interconnects minimizes parasitic capacitance and displacement currents by maintaining conductors at the same potential as the sensitive node. This approach enhances common-mode rejection and noise performance without requiring higher supply voltages. Signal averaging techniques can further reduce random noise by lowering the root-mean-square (RMS) error through repeated measurements [125]. However, this approach introduces a trade-off between noise reduction and temporal resolution, as averaging may obscure fast transient electrochemical events. The noise performance in potentiostat systems is governed by the entire signal chain, from input-stage amplification to filtering, shielding, and post-processing. Trade-offs between noise suppression, bandwidth, and temporal resolution ultimately define the minimum detectable signal and the accuracy of electrochemical measurements. Thus, noise mitigation strategies set the fundamental limits on sensitivity and dynamic response in electrochemical sensing systems.

Noise performance and current resolution fundamentally shape circuit-level design strategies, including the use of low-noise operational amplifiers, chopper stabilization, and mismatch-tolerant architectures to enable picoampere-level detection. Achieving such low noise in miniaturized systems requires careful bandwidth control within a defined power–resolution trade-off. Increasingly, these hardware advances are complemented by software-assisted protocols, including scripting and adaptive calibration, to enhance reproducibility and system-level control rather than replace hardware optimization. These design considerations, along with associated power consumption trade-offs, are summarized in Table 2 across representative potentiostat platforms.

### 4.8. Control Software and User Interfaces

Control software provides the interface between the user, internal electronics, and the electrochemical cell, enabling configuration and execution of potentiostatic experiments. It defines experimental parameters such as waveform type, potential limits, timing, and data acquisition settings, which govern electrochemical boundary conditions and ensure synchronization between signal generation and measurement [125]. In research-grade systems, control software supports multiple electrochemical techniques with customizable protocols. For example, EC-Lab^®^ (BioLogic) V11.50 to V11.60 (Seyssinet-Pariset, France) provides built-in methods with adjustable parameters for applied potential or current, timing, and temperature, enabling stable and reproducible operation of the control amplifier, feedback loop, and analog front end (AFE) [125,126].

Advanced software platforms enable real-time modification of experimental parameters. Features such as EC-Lab’s Modify-on-the-Fly allow dynamic adjustment of voltage, current, scan rate, and timing during operation while maintaining synchronization with the acquisition system [127]. This capability supports adaptive experimentation, enabling correction of transient effects and optimization of measurement conditions. Automation and data processing are integral to modern potentiostat software. Platforms such as PSTrace (PalmSens) support scripting for automated workflows, including sequential measurements and batch analysis [128,129]. These systems incorporate filtering and preprocessing pipelines to improve data quality and enable export to external tools such as Excel, MATLAB, and Origin [129,131]. Graphical programming environments, such as LabVIEW, further enable integration of control logic, signal processing, and hardware interfacing within a unified platform [130].

User interfaces (UIs) provide interactive access to potentiostat functions through graphical displays for real-time monitoring and control. Modern interfaces enable visualization of electrochemical responses, adjustment of experimental parameters, and navigation across multiple techniques within a structured environment [132,133]. Interactive features, such as dynamic tuning of scan rate, potential limits, and timing, enhance system flexibility and support responsive control of electrochemical processes [134]. Hardware–software integration enables coordinated operation across multiple instruments and supports remote monitoring over local area networks (LAN), as demonstrated in systems such as the VSP-series potentiostats [135,136]. Overall, control software and user interfaces govern synchronization, parameter definition, and data processing across the potentiostat system. Their integration with hardware enables adaptive control, automation, and real-time visualization, which collectively determine measurement accuracy, reproducibility, and scalability in modern electrochemical sensing platforms. Thus, software architecture defines the functional limits of modern potentiostatic systems, bridging hardware performance with analytical capability.

## 5. Communication Protocols in Potentiostats

Communication protocols define the rules and procedures that enable data exchange between the potentiostat and external systems such as computers, microcontrollers, and mobile devices. These protocols support transmission of control commands (e.g., experiment initiation, parameter adjustment) and acquisition of electrochemical data (e.g., current, voltage, time, impedance) [134,137]. Reliable communication is essential for maintaining synchronization between signal generation and data acquisition, ensuring accurate timing and measurement fidelity.

### 5.1. Internal Communication Protocols

Internal protocols facilitate communication between system components such as microcontrollers, DACs, ADCs, and AFE circuits. The inter-integrated circuit (I^2^C) is a low-speed, two-wire protocol using serial data (SDA) and clock (SCL) lines [138,139]. It is well-suited for configuration tasks such as setting gain values, controlling programmable amplifiers, and adjusting reference circuitry. Its addressing capability allows multiple devices to share a single bus, reducing wiring complexity in compact systems. However, its limited data rate restricts its use to control and configuration rather than high-speed data transfer.

The serial peripheral interface (SPI) is a synchronous protocol employing multiple lines (MOSI, MISO, clock, chip select) to enable high-speed, full-duplex communication [140,141,142,143,144]. SPI supports rapid data exchange between DACs, ADCs, and control units, providing deterministic timing and low-latency updates required for fast-scan cyclic voltammetry and high-frequency EIS [141,142].

### 5.2. External Communication Interfaces

External protocols connect potentiostats to user interfaces and host systems. Universal asynchronous receiver–transmitter (UART) provides a simple asynchronous interface using transmit (TX) and receive (RX) lines [145,146]. It is commonly used in low-cost systems for command transmission and moderate-rate data streaming, offering reliability with limited bandwidth. Legacy standards such as RS-232 and RS-485 define electrical signaling for serial communication. RS-232 uses bipolar signaling and has been widely adopted in laboratory systems due to its simplicity [147,148]. RS-485 extends communication over long distances (>100 m) using differential signaling, significantly improving noise immunity and enabling reliable operation in electrically noisy or distributed sensing environments [149,150,151].

Universal Serial Bus (USB) has become a dominant wired interface due to its high bandwidth, plug-and-play capability, and integrated power delivery [152,153,154]. USB-based potentiostats enable simultaneous power and data transfer, simplifying system architecture while supporting high-throughput measurements comparable to benchtop instruments [62,155,156]. This approach falls within wired interface architectures, which provide low latency, moderate power consumption, and high data integrity, but inherently constrain system portability.

### 5.3. Wireless Communication Protocols

Wireless communication enhances portability and flexibility in potentiostat systems. Near-field communication (NFC) enables ultra-low-power, short-range (~4 cm) communication through magnetic coupling, allowing battery-less operation powered by external devices such as smartphones [157,158,159]. This makes NFC suitable for disposable and point-of-care sensing applications [160,161,162]. Power fluctuations inherent to NFC systems necessitate multi-layer stabilization strategies, including rectification of harvested energy, intermediate buffering to mitigate short-term variations, and regulation via low-dropout (LDO) circuits to establish stable supply rails and reference potentials. Bandgap-derived references and energy buffering further stabilize voltage behavior, while undervoltage lockout and power-good gating prevent erroneous measurements by suspending operation below defined thresholds. For wireless communication, Bluetooth Low Energy (BLE) offers low-power, secure data transfer with frequency hopping and AES-128 CCM encryption at the link layer, enabling efficient real-time monitoring and control via mobile devices in wearable potentiostats [163,164,165,166,167]. In contrast, Wi-Fi provides higher bandwidth and extended range, supporting cloud connectivity and browser-based interfaces (e.g., ESP32 platforms), but at the cost of increased power consumption and latency [134,168,169,170]. Wi-Fi security relies on WPA2/WPA3 protocols with AES-based hardware encryption, supplemented by transport- and application-layer protections. These trade-offs highlight the need to balance power stability, communication reliability, and security, particularly in resource-constrained wearable sensing systems.

### 5.4. Cloud and IoT Integration

Modern potentiostats increasingly incorporate cloud-based communication using protocols such as HTTP for remote data storage, access, and analysis [171,172,173]. These systems support real-time monitoring, automated logging, and integration with machine learning workflows. Platforms such as FreiStat demonstrate the feasibility of cloud-connected electrochemical sensing with distributed analytical capabilities [145]. These communication protocols define the temporal coordination, data throughput, and system scalability of potentiostat operation. Trade-offs between speed, power consumption, noise immunity, and range determine the suitability of each protocol for specific functions, from high-speed internal control to long-range or low-power wireless sensing. Thus, communication architecture defines the limits of synchronization, data integrity, and scalability in modern potentiostat systems. Local (edge) processing reduces data transmission and power consumption but constrains algorithmic complexity, whereas cloud-based processing enables advanced analytics at the cost of latency and connectivity dependence. As a result, hybrid architectures are increasingly favored, where lightweight edge processing supports calibration, anomaly detection, and feature extraction, while computationally intensive analysis and long-term data aggregation are delegated to cloud infrastructures.

Table 3 summarizes platforms spanning diverse applications, from low-cost educational devices to cloud-integrated biosensing and long-term corrosion monitoring. Rather than providing a simple ranking of individual designs, the comparison highlights key architectural trade-offs that govern performance, scalability, and deployment. Importantly, this synthesis emphasizes reported and qualitative benchmarks, reflecting the current lack of standardized evaluation metrics across studies, rather than relying solely on strictly normalized performance values.

## 6. Challenges of Potentiostats

Electrochemical sensing is increasingly transitioning from benchtop instruments to compact, portable, wearable, and wireless potentiostats. Achieving comparable performance in these systems requires operation within constrained voltage compliance, current range, and bandwidth imposed by size and power limitations. For example, the smartphone-enabled Universal Wireless Electrochemical Detector (UWED) demonstrated benchtop-like performance across potentiometry, CV, CA, and square wave voltammetry (SWV) within a potential range of ±1.5 V and a current range of ±180 µA, yet exhibits reduced hardware robustness and long-term stability compared to laboratory systems. Similarly, battery-less NFC-based potentiostats operate within narrower ranges (±0.8 V, ±20 µA), limiting their applicability in high-throughput or long-duration measurements.

AFE integration presents additional challenges. While integrated solutions such as ADuM355 enable advanced techniques, achieving low-noise current detection in the nanoampere to picoampere range under battery operation requires careful shielding, thermal stabilization, and calibration. In compact systems, noise, thermal drift, and bias instability are amplified by low-power constraints, limiting high-sensitivity measurements. Wireless communication further complicates system design. Bluetooth Low Energy (BLE) supports low-power operation and seamless integration with mobile devices but may require data compression for high-density measurements such as CV, SWV, and EIS. In contrast, Wi-Fi enables high-throughput data streaming and cloud connectivity at the cost of increased power consumption. Integration of multiple communication protocols (e.g., USB, BLE, Wi-Fi) introduces additional challenges in firmware scheduling, antenna placement, and ground plane design, which can degrade analog front-end performance due to electromagnetic interference.

Ensuring reproducibility and equivalence to benchtop systems remains a critical challenge. Platforms such as TBISTAT and FreiStat have demonstrated performance comparable to laboratory instruments for techniques such as EIS, CV, and differential pulse voltammetry (DPV). However, consistent performance across electrode batches, environmental conditions, and sample matrices remains difficult to achieve, particularly in field-deployed or clinical applications. Power and thermal constraints further limit continuous operation. High-duty-cycle wireless communication, local processing, and extended measurements such as EIS sweeps increase power consumption and thermal load, which can elevate noise floors and degrade measurement stability. These effects are amplified in multi-channel systems with higher computational demands.

Modern potentiostat designs must also balance on-device computation, data transmission, and storage. Tasks such as buffering, preprocessing, and delayed cloud synchronization introduce latency and resource constraints, particularly in systems operating under intermittent connectivity. The primary challenge in modern potentiostat design lies in balancing miniaturization, sensitivity, power consumption, and connectivity. Constraints in voltage range, noise performance, communication bandwidth, and thermal stability are tightly coupled, and optimization of one parameter often degrades another, defining fundamental trade-offs in portable and distributed electrochemical sensing systems. These constraints define the fundamental design space for next-generation potentiostats and guide the development of optimized architectures for portable and wearable sensing.

## 7. Future Prospect of Potentiostats

Future potentiostat development is expected to emphasize seamless integration of hardware, software, and communication systems to enable accessible, scalable, and high-performance electrochemical sensing. Cloud-based architectures are increasingly enabling app-independent operation, where experimental control, data processing, and visualization are performed through standard-compliant web interfaces. These systems allow multiple users, including clinicians, caregivers, and patients to access measurements through browser-based platforms without dedicated software installations.

Communication protocols are expected to evolve toward purpose-specific implementations rather than general-purpose connectivity. Lightweight protocols such as MQTT are well-suited for low-latency, bidirectional control and telemetry, while HTTPS-based frameworks support secure remote experimentation and data transfer. Edge advances–cloud co-design will enable adaptive sampling, event-driven data transmission, and efficient bandwidth utilization while preserving measurement fidelity. Systems such as FreiStat demonstrate this approach by integrating embedded computation with cloud orchestration to enable continuous analysis without increasing hardware complexity. Hierarchical processing is likely to be adopted with the architectural impact on the ADC resolution, sampling rate and bandwidth based on the relevance of data required. Additionally, local processing blocks will be useful in performing feature extraction, event detection and averaging followed by wireless transmission.

Open and validated architectures are expected to play a central role in improving reproducibility and accelerating adoption. Continued development of standardized firmware, hardware platforms, and benchmarking procedures, such as those associated with FreiStat and TBISTAT will facilitate cross-platform validation and regulatory acceptance across applications including biosensing and corrosion monitoring. Battery-less and ultra-low-power systems, particularly those enabled by NFC, are anticipated to expand from passive sensing to active electrochemical monitoring. These platforms enable compact, field-deployable, and wearable devices capable of periodic screening and adherence monitoring. Emerging sensing strategies, such as nanozyme-based assays for biomarkers, demonstrate the feasibility of clinically relevant measurements without the need for high power consumption or complex fluid handling.

Integration of artificial intelligence (AI) and machine learning (ML) is expected to enhance the reliability of electrochemically measured signals. Current implementations include calibration and classification algorithms to improve repeatability and reduce user intervention. Future systems are likely to incorporate tight coupling between AFE and digital processing blocks to ensure anomaly detection and support adaptive waveform generation, and context-aware signal interpretation, enabling dynamic optimization of measurement conditions. Lightweight interference-only will also drive moderate on-chip memory, low-latency data paths and efficient numerical processing without necessarily having high-performance processors. Interoperability with broader digital ecosystems will become increasingly important. Integration with wireless communication frameworks and secure data infrastructures will support decentralized sensing, enabling seamless transmission of measurements from home or field environments to clinical or analytical dashboards.

CMOS integration and system-on-chip (SoC) architectures are expected to increasingly incorporate discrete potentiostats to enable multi-analyte detection through programmable configurations. While such integration offers advantages in power efficiency, device miniaturization, and reproducibility, it comes at the cost of reduced post-fabrication flexibility. In wearable and flexible platforms, performance is further constrained by motion-induced noise, variable electrode impedance, and mechanical deformation. Addressing these challenges will require motion-tolerant front-end electronics (AFE), including differential sensing, shielding, and bandwidth optimization, prioritizing robustness over absolute sensitivity. Consequently, modular or distributed system architectures may emerge, separating the electrochemical interface from processing units to enhance mechanical reliability and wearability. Overall, continued advancements are likely to drive convergence between sensing, processing, and connectivity, positioning the potentiostat as an integrated component rather than an isolated measurement module.

The future of potentiostats lies in the convergence of cloud-native architectures, intelligent signal processing, standardized platforms, and energy-efficient hardware. This integration enables scalable, adaptive, and networked electrochemical sensing systems, where trade-offs between power, data throughput, and measurement fidelity are optimized through coordinated hardware–software-communication co-design. This paradigm shift positions potentiostats as core components of future intelligent sensing infrastructures rather than standalone analytical tools.

## 8. Conclusions

Advances in potentiostat technology have transformed electrochemical systems, enabling precise control and measurement of redox processes across diverse applications. The evolution from analog instrumentation to miniaturized and integrated systems reflects parallel progress in electronics, signal processing, and embedded computing. This review examined key operational principles, including OPAs, AFE architectures, and signal generation and acquisition, emphasizing their integration with digital control systems. A central outcome is that modern potentiostat performance is no longer determined solely by analog circuitry, but by the coordinated interaction of hardware, communication protocols, and data-processing frameworks. The shift toward wireless and cloud-enabled architectures has expanded accessibility and enabled real-time electrochemical sensing through smartphone and web-based platforms.

However, achieving benchtop-level precision in portable systems remains constrained by trade-offs in power consumption, noise, thermal stability, and bandwidth, particularly for low-current (nA–pA) measurements. Reproducibility across varying conditions and robust integration of communication and data infrastructure also remain critical challenges. Looking forward, potentiostats are converging toward decentralized, intelligent sensing systems through integration with edge computing, cloud platforms, and machine learning. These advances enable adaptive measurement strategies, automated analysis, and scalable deployment in point-of-care, environmental, and industrial applications. The key paradigm shift is from standalone measurement devices to connected, system-level sensing nodes, where performance is defined by the co-design of electronics, communication, and data intelligence. As this convergence advances, potentiostats will remain foundational to next-generation electrochemical sensing, supporting ubiquitous, real-time, and distributed analytical systems.

## Figures and Tables

**Table 1:** Summary of performance range of the SAR and Sigma-Delta DACs.

Parameter	ADCs	
	SAR	Sigma-Delta
Typical Resolution	10–14 bits	16–24 bits
Effective Number of Bits	9–12 bits	15–20 bits
Sampling/Output Rate	100 kS/s–1 MS/s	1 S/s–10 kS/s
Power Consumption	10–500 µW	50 µW–2 mW
Energy per conversion	~10–100 pJ	~1–100 nJ
Latency	<1 µS	0.1–10 ms

**Table 2:** Representative Quantitative Benchmarks for Modern Potentionstat Architectures.

Architecture/Context	Current Resolution/Noise Floor	SNR	Power Consumption	Physical Scale	Representative References
Laboratory potentiostats with advanced software control	<10–100 fA	>90 dB	>1 W	Instrument Scale	[120,125,126,127]
Discrete low-noise AFE designs	10–1000 fA	70–90 dB	100–500 mW	PCB-Scale	[121,123]
Chopper-stabilizedmengye/Mismatch-tolerant AFEs	10–10^4^ fA	50–85 dB	1–50 mW	cm^2^ scale	[123,124]
Miniaturized biomedical signal acquisition systems	~1–10 pA (effective)	50–75 dB	<1–10 mW	Compact/wearable	[122,124]
Scripted/software-assisted reproducible platforms	Hardware-dependent	Hardware-dependent	Platform-dependent	Bench-top/portable	[125,126,127,128,129]
Portable EIS-capable systems	Portable EIS-capable systems	Technique-dependent	10–100 mW	Portable	[130]

**Table 3:** Comparison of Representative Portable and Wireless Potentiostat Platforms.

Ref.	System Type	Wireless Interface	Power Consumption	Approximate Footprint	Wireless Throughput/Usage
[169]	Low-cost portable	BLE	Battery-Powered (mW range)	Handheld PCB-scale	Low-rate streaming to a mobile device
[170]	Reconfigurable AFE platform	Wireless (reconfigurable)	Portable, duty-cycled	Compact modular system	Adjustable data rates for POCT and monitoring
[171]	Remote monitoring system	Long-range wireless	Optimized for long-term (3.8 years)	Field-deployable unit	Low-bandwidth telemetry
[172]	Cloud-connected portable	Wi-Fi	Higher power (ESP-32-class)	Embedded compact system	Continuous data streaming to the cloud
[173]	Low-cost portable	Wireless	Battery-powered	Small PCB-based device	Real-time data sharing at modest bandwidth

## Data Availability

No new data were created or analyzed in this study. Data sharing is not applicable to this article.
